# HJ11 decoction restrains development of myocardial ischemia-reperfusion injury in rats by suppressing ACSL4-mediated ferroptosis

**DOI:** 10.3389/fphar.2022.1024292

**Published:** 2022-11-22

**Authors:** Fangyuan Zhang, Ziyun Li, Ping Gao, Jiaxi Zou, Yuting Cui, Yi Qian, Renjun Gu, Weiming Xu, Jingqing Hu

**Affiliations:** ^1^ Institute of Basic Theory for Chinese Medicine, China Academy of Chinese Medical Sciences, Beijing, China; ^2^ School of Acupuncture and Tuina, School of Regimen and Rehabilitation, Nanjing University of Chinese Medicine, Nanjing, China; ^3^ Affiliated Hospital of Integrated Traditional Chinese and Western Medicine, Nanjing University of Chinese Medicine, Nanjing, China; ^4^ School·of·Basic·Medical·Sciences Chengdu·University·of Traditional·Chinese Medicine, Chengdu, China; ^5^ College of Chinese Medicine, Changchun University of Chinese Medicine, Changchun, Jilin, China; ^6^ The Third School of Clinical Medicine, Nanjing University of Chinese Medicine, Nanjing, China; ^7^ School of Chinese Medicine, School of Integrated Chinese and Western Medicine, Nanjing University of Chinese Medicine, Nanjing, China; ^8^ China Science and Technology Development Center for Chinese Medicine, Beijing, China; ^9^ The First Affilliated Hospital of Henan University of CM, Zhengzhou, China

**Keywords:** HJ11 decoction, myocardial I/R injury, ferroptosis, oxidative stress, cardiac function

## Abstract

HJ11 is a novel traditional Chinese medicine developed from the appropriate addition and reduction of Si-Miao-Yong-An decoction, which has been commonly used to treat ischemia-reperfusion (I/R) injury in the clinical setting. However, the mechanism of action of HJ11 components remains unclear. Ferroptosis is a critical factor that promotes myocardial I/R injury, and the pathophysiological ferroptosis-mediated lipid peroxidation causes I/R injury. Therefore, this study explored whether HJ11 decoction ameliorates myocardial I/R injury by attenuating ACSL4-mediated ferroptosis. This study also explored the effect of ACSL4 expression on iron-dependent programmed cell death by preparing a rat model of myocardial I/R injury and oxygen glucose deprivation/reperfusion (OGD/R)–induced H9c2 cells. The results showed that HJ11 decoction improved cardiac function; attenuated I/R injury, apoptosis, oxidative stress, mitochondrial damage, and iron accumulation; and reduced infarct size in the myocardial I/R injury rat model. Additionally, HJ11 decoction suppressed the expression of ferroptosis-promoting proteins [Acyl-CoA synthetase long-chain family member 4 (ACSL4) and cyclooxygenase-2 (COX2)] but promoted the expression of ferroptosis-inhibiting proteins [ferritin heavy chain 1 (FTH1) and glutathione-dependent lipid hydroperoxidase glutathione peroxidase 4 (GPX4)] in the myocardial tissues of the I/R injury rat model. Similar results were found with the OGD/R-induced H9c2 cells. Interestingly, ACSL4 knockdown attenuated iron accumulation, oxidative stress, and ferroptosis in the OGD/R-treated H9c2 cells. However, ACSL4 overexpression counteracted the inhibitory effect of the HJ11 decoction on OGD/R-triggered oxidative stress and ferroptosis in H9c2 cells. These findings suggest that HJ11 decoction restrained the development of myocardial I/R injury by regulating ACSL4-mediated ferroptosis. Thus, HJ11 decoction may be an effective medication to treat myocardial I/R injury.

## Introduction

Acute myocardial infarction (AMI) is one of the leading causes of disability and death globally and imposes severe burden on health and economy (Altshuler et al., 2021). More than seven million patients suffer from AMI every year, and untreated AMI cases contribute to approximately 30% of all AMI-related deaths (Beom et al., 2021). The widely used treatment strategy for AMI aims to restore blood flow in a timely manner through percutaneous coronary intervention (Chen et al., 2021a). This treatment method ensures safety and efficacy in the clinical setting, but the ischemia/reperfusion (I/R) process can induce irreversible damage to the myocardial tissues (a condition known as myocardial I/R injury) (Chouchani et al., 2016). Myocardial I/R injury is an unavoidable adverse event that occurs in AMI treatment (Cui et al., 2021). The common adverse symptoms induced by myocardial I/R injury are arrhythmia, deterioration of cardiac function, and myocardial tissue damage (Denorme et al., 2020; Di et al., 2021). An effective adjuvant therapy administered to alleviate myocardial I/R injury can improve the clinical prognosis of AMI patients (Ding et al., 2020). However, there is an urgent need to discover effective treatment strategies for myocardial I/R injury.

Different from necrosis and apoptosis, ferroptosis is a newly discovered form of cell death, that is, iron-dependent programmed cell death. Ferroptosis occurs in multiple diseases, such as malignant tumors, neurodegenerative diseases, and cardiovascular diseases (Denorme et al., 2020). Mitochondrial damage (characterized by shrinkage of the mitochondria and broken mitochondrial cristae), reactive oxygen species (ROS) accumulation, and lipid peroxidation, all of which are induced by ferroptosis, are the main reasons for I/R injury in multiple organs such as the brain and liver (Doll et al., 2017; Dong et al., 2020). Recently, ferroptosis has been identified as a key risk factor for the induction of myocardial I/R injury. Acyl-CoA synthetase long-chain family member 4 (ACSL4) is a lipid metabolism enzyme that is an essential component for the execution of ferroptosis (Eefting et al., 2004; Fan et al., 2021). Anti-ferroptosis agents and ACSL4 exacerbate ferroptosis after myocardial I/R injury (Fu et al., 2021).

Some of the compounds used in traditional Chinese medicine possess ferroptosis-suppressing property. For instance, naringenin is a flavonoid that is abundant in fructus aurantia and rhizoma drynariae, and it attenuates ferroptosis to alleviate myocardial I/R injury (He et al., 2017). Resveratrol is an extract of polygonum cuspidatum and has demonstrated effects similar to those of naringenin (Hu et al., 2021). Additionally, cyanidin-3-glucoside is a member of the anthocyanin family and is abundant in red or purple fruits and vegetables. It inhibits ferroptosis to ameliorate myocardial I/R injury (Denorme et al., 2020). Baicalin, a Chinese medicine isolate from Scutellaria root, mitigates myocardial I/R injury by attenuating ferroptosis through the inhibition of ACSL4 expression (Huang et al., 2019). It is well known that naringenin, resveratrol, cyanidin-3-glucoside, and baicalin are traditional herbal ingredients that are extracted from different plants. The aforementioned information illustrates the effectiveness of traditional Chinese medicine in the treatment of myocardial I/R injury through the ferroptosis-suppressing effect. However, more effective traditional Chinese medicines need to be discovered to provide more effective treatment regimens for myocardial I/R injury. Si-Miao-Yong-An decoction can effectively improve heart function, reduce the extent of damage due to cardiovascular disease, and alleviate myocardial I/R injury (Jiang et al., 2021; Jin et al., 2021). HJ11 decoction is developed from appropriate addition and reduction of Si-Miao-Yong-An decoction and has been commonly used to treat I/R injury in the clinical setting. It is approved by the Patent Office of China for the invention (patent application 2110658 CN), and a clinical trial (CHiCTR2100043038) has been conducted simultaneously. HJ11 decoction contains not only the components of Si-Miao-Yong-An decoction, such as Lonicerae japonicae flos and Scrophulariae Radix (Jing et al., 2021), but also some new components. The components of HJ11 decoction are in the confidential stage. This study aimed to verify whether HJ11 decoction restrains myocardial I/R injury by suppressing ACSL4-mediated ferroptosis. Additionally, the effect of ACSL4 expression on iron-dependent programmed cell death was studied by preparing a rat model of myocardial I/R injury (*in vivo* animal studies) and oxygen glucose deprivation/reperfusion (OGD/R)–induced H9c2 cells (*in vitro* molecular mechanism studies). The findings of this research showed that HJ11 decoction could attenuate myocardial I/R injury by inhibiting ACSL4-mediated ferroptosis. Thus, HJ11 decoction may be an effective herbal medicine for treating myocardial I/R injury.

## Materials and methods

### Preparation of HJ11 decoction

Chinese Herbal Pieces for HJ11 decoction were provided by CR SANJIU Pharmaceutical Co., Ltd. (Hefei, Anhui, China; Chinese Pharmacopoeia, 2015). The HJ11 decoction was prepared according to the following process: The Chinese Herbal Pieces (weight: 100 g) were boiled in 500 g of distilled water for 50 min to collect the decoction by filtration. Then, 300 g of distilled water was added to the remaining dregs, and the solution was boiled for 50 min. The decoction was collected again by filtration. The two decoction solutions were mixed together and then concentrated to a final concentration of 4.5 g/ml under boiling conditions. The prepared HJ11 decoction was stored at −20°C for subsequent use.

### Baseline toxicity testing of HJ11 decoction

Six male adult Sprague Dawley rats (weight: 200 ± 20 g; Charles River Laboratories, Beijing, China) received a high dose (18 g/kg/d) of the HJ11 decoction through gavage. After 28-day consecutive administration, the rats were subjected to echocardiographic analysis to evaluate cardiac function. The rats were then euthanized by deep anesthetization with inhalation of 5% isoflurane. The whole heart, liver, spleen, lung, and kidney were obtained, and the extent of damage was assessed by hematoxylin-eosin (HE) staining. The animal experiments were conducted after ratification by the Animal Ethics Committee and were performed in line with the Guide for the Care and Use of Laboratory Animals.

### Construction of a myocardial I/R injury rat model and treatment with HJ11 decoction

Sixty male adult Sprague Dawley rats (weight: 200 ± 20 g) were commercially purchased from Charles River Laboratories (Beijing, China). The rats were kept in a specific pathogen-free room at 20°C–22°C, relative humidity of 55%–65%, and 12-h day/night cycle. The rats had free access to food and water. After 7 days of rearing, the rats underwent myocardial I/R surgery for adaptation to the environment. The animal experiment was conducted after ratification by the Animal Ethics Committee and was performed in line with the Guide for the Care and Use of Laboratory Animals.

All 60 rats were randomly divided into five groups: Sham group, I/R group, I/R + HJ11-L group, I/R + HJ11-M group, and I/R + HJ11-H group. Myocardial I/R surgery was performed on rats of the I/R group, I/R + HJ11-L group, I/R + HJ11-M group, and I/R + HJ11-H group. The following procedure was used: Rats of the four groups were anesthetized by inhalation of 5% isoflurane, and the anesthesia was maintained by inhalation of 2% isoflurane (Jung et al., 2021). After ensuring that the rats did not respond to limb stimulation, they were fixed on the operating table in the supine position. The rats were placed in an animal ventilator, and routine skin disinfection of the surgical area was performed. Thoracotomy was performed between the third and fourth intercostal space on the left side to expose the heart. The proximal left anterior descending coronary artery was then ligated with a 6.0-gauge surgical suture. The appearance of whitening of the ligated part of the myocardium indicated that the myocardial ischemia was effective. After 60 min of ischemia, the ligature was removed to restore myocardial blood supply to complete the reperfusion process. Finally, the rats were placed in sterile cages after chest closure. For rats of the Sham group, only thoracotomy and threading (without ligation) were performed.

The next day after surgery, rats of the I/R + HJ11-L group, I/R + HJ11-M group, and I/R + HJ11-H group received HJ11 decoction through gavage at the following doses: 4.5, 9, and 18 g/kg/d, respectively. HJ11 decoction was administered for 28 consecutive days. Rats of the Sham group and I/R group received an equal volume of distilled water for 28 consecutive days.

### Echocardiographic analysis

After treatment with HJ11 decoction for 28 days, rats of each group were subjected to echocardiographic analysis using Vevo3100 with an MX250 probe (FUJIFILM VisualSonics, Inc., Toronto, ON, Canada) at an ultrasound frequency of 21 MHz. Before echocardiographic analysis, the rats were anesthetized by inhalation of 2% isoflurane and placed in the supine position on a thermal blanket at 37°C. Long-axis B-ultrasound and M-ultrasound images of the heart were obtained by performing ultrasound examination in the left parasternal region. Then, the MX250 probe was rotated 90° to acquire short-axis B-ultrasound and M-ultrasound images at the papillary muscle level. Short-axis M-ultrasound images of the left ventricular region were performed to measure the left ventricular end-diastolic diameter (LVEDd), left ventricular ejection fraction (LVEF), left ventricular end-systolic diameter (LVEDs), and left ventricular fractional shortening (LVFS) of rats. All ultrasound measurements were performed over three consecutive cardiac cycles to calculate the average values.

### Euthanasia of rats and collection of heart and serum

After treatment with HJ11 decoction for 28 days, blood samples were obtained from the abdominal aorta. The rats were then deeply anesthetized by inhalation of 5% isoflurane. When the rats did not respond to limb stimulation, they were killed by rapid cervical dislocation, and the whole heart of each rat was obtained.

### Hematoxylin-eosin staining

HE staining was performed to evaluate the extent of injury to the myocardial, liver, spleen, lung, and kidney tissues. Briefly, these tissues were washed with phosphate-buffered saline (PBS). For rats with myocardial I/R injury, the myocardial tissues in the I/R injury region were used for detection. After fixing the myocardial tissues with 4% paraformaldehyde for 48 h, these tissues were embedded into paraffin and cut into 4-µm-thick sections. The sections were then immersed into xylene and gradient alcohol with a series of descending concentrations for dewaxing and rehydration. Hematoxylin staining solution (Solarbio, Beijing, China) was added onto the rehydrated sections for staining for 5 min at room temperature. The residual staining solution was rinsed off under tap water. The sections were then treated with 1% hydrochloric acid ethanol solution for 1 min for their differentiation, followed by staining with eosin staining solution (Solarbio, Beijing, China) for 1 min at room temperature. The sections were washed under tap water to remove the residual staining solution. The stained sections were treated with xylene and gradient alcohol with a series of descending concentrations for dehydration. Finally, the sections were sealed in neutral resin and observed under an optical microscope (Olympus, Tokyo, Japan).

### Terminal deoxynucleotidyl transferase uridyl nick-end labeling assay

The apoptosis of cardiomyocytes in the myocardial tissues was assessed using the terminal deoxynucleotidyl transferase dUTP nick end labeling (TUNEL) assay kit (Roche Diagnostics, Shanghai, China), according to the manufacturer’s instructions. Briefly, the myocardial tissue sections were prepared in the same way as that described in the “HE staining” section. After the routine dewaxing and hydration processes, the sections were permeabilized with 0.25% Triton-X 100 for 20 min (Beyotime, Shanghai, China). After reaction with the TUNEL reaction mixture for 1 h, the sections were stained with the hematoxylin staining solution (Solarbio, Beijing, China) for 5 min. After dehydration and sealing, the sections were observed under an optical microscope (Olympus, Tokyo, Japan) and photographed. TUNEL-positive cells were counted using Image-Pro Plus 6.0 software (Media Cybernetics, Inc., Rockville, MD, United States). The rate of TUNEL-positive cells was calculated as follows: (number of TUNEL-positive cells/total number of cells) × 100%.

### Triphenyl tetrazolium chloride staining

The whole heart of rats was cut into 1.5-mm-thick pieces parallel to the coronal sulcus. The pieces were then incubated with 2,3,5-triphenyltetrazolium chloride (TTC) staining solution (Boster, Wuhan, China) for 15 min in the dark. The infarct area was exhibited as white, and the noninfarct area was presented as an area. The size of the infarct area and the noninfarct area was analyzed using Image-Pro Plus 6.0 software. The rate of the infarct area was calculated as follows: infarct area/(infarct area + noninfarct area).

### Prussian blue staining

The myocardial tissues in the I/R region were prepared in the same way as that described in the “HE staining” section. The routine processes of dewaxing and rehydration of the sections were then carried out. The sections were stained with the Prussian blue staining solution (Solarbio, Beijing, China) for 40 min at room temperature. The residual staining solution was removed by washing the sections with distilled water. The sections were then stained with the hematoxylin staining solution (Solarbio, Beijing, China) for 5 min. The sections were washed under tap water and then routinely dehydrated. The stained sections were sealed in neutral resin, following which they were observed under an optical microscope (Olympus, Tokyo, Japan) and photographed. The number of cells with Prussian blue–positive staining was evaluated using Image-Pro Plus 6.0 software. The rate of cells with Prussian blue−positive staining was determined as follows: (number of cells with Prussian blue−positive staining/total number of cells) × 100%.

### Detection of creatine kinase isoenzyme and lactate dehydrogenase

The levels of creatine kinase isoenzyme (CK-MB) and lactate dehydrogenase (LDH) in the myocardial tissues of the I/R region of rats were tested using the CK-MB Kit (Jianglai Biotechnology Co., Ltd., Shanghai, China) and the LDH Kit (Kanglang Biotechnology Co., Ltd., Shanghai, China), respectively. The myocardial tissues from the I/R region of rats were homogenized on ice, followed by centrifugation for 10 min at 1,000 × g and 4°C to collect the supernatant. The detection of the CK-MB and LDH levels in the supernatant was performed strictly according to the manufacturers’ instructions.

### H9c2 cell culture

H9c2 cells were purchased from Fuheng Biotechnology Co., Ltd. (FH1004, Shanghai, China). The cells were fostered in Dulbecco’s Modified Eagle Medium (DMEM; Solarbio, Beijing, China) supplemented with 10% fetal bovine serum (FBS; Solarbio, Beijing, China), 100 U/ml penicillin (Solarbio, Beijing, China), and 100 U/mL streptomycin (Solarbio, Beijing, China) at 37°C, 5% CO_2_, and 95% air. The medium was replaced every 3 days.

### Oxygen glucose deprivation/reperfusion and HJ11 decoction and ferrostatin-1 treatment to H9c2 cells

The OGD/R experiment is a commonly used method for establishing the *in vitro* I/R cell model, which includes two steps: the first step is the establishment of ischemic injury by treating cells with a low-glucose and -serum medium under a hypoxic state; the second step is the construction of reperfusion by treating cells with a normal-glucose and -serum medium under a normoxic state. This process is similar to the I/R process *in vivo* (Lee et al., 2018). In this study, OGD/R treatment was applied on H9c2 cells to construct the myocardial I/R injury cell model. H9c2 cells were first exposed to the hypoxic condition (1% O_2_, 5% CO_2_, and 94% N_2_) for 24 h in a low-glucose and -serum DMEM, followed by exposure to the reoxygenation condition (5% CO_2_ and 95% air) for 12 h in high-glucose DMEM supplemented with 10% FBS (Lei et al., 2020).

HJ11 decoction treatment was applied after OGD/R treatment. The H9c2 cells were cultured in DMEM supplemented with 10% FBS and different concentrations of HJ11 decoction (100, 200, 400, 600, 800, and 1,000 μg/ml) at 37°C, 5% CO_2_, and 95% air.

Moreover, after OGD/R treatment, H9c2 cells were cultured in DMEM supplemented with 10% FBS and ferrostatin-1 [Fer-1, ferroptosis inhibitor, 10 µM (Lesnefsky et al., 2017)] at 37°C, 5% CO_2_, and 95% air.

### Transfection of H9c2 cells

H9c2 cells were harvested at nearly 85% confluence, dispersed into single-cell suspension with serum-free DMEM (1 × 10^6^ cells/ml), and seeded into 6-well plates containing 1 ml of the cell suspension per well. Small interfering RNA (siRNA) targeting ACSL4, siRNA negative control (NC), pcDNA3.1-ACSL4 expression vectors, and pcDNA3.1 empty vectors were commercially purchased from GeneChem (Shanghai, China). These vectors were individually transfected into H9c2 cells in accordance with the instructions of Lipofectamine 3000 (Thermo Fisher Scientific, Shanghai, China). After transfection, H9c2 cells were maintained in DMEM supplemented with 10% FBS and cultured at 37°C, 5% CO_2_, and 95% air for 48 h. To determine transfection efficiency, H9c2 cells were collected and subjected to real-time quantitative reverse transcription–polymerase chain reaction (qRT-PCR).

Moreover, the OGD/R-induced H9c2 cells were transfected by pcDNA3.1-ACSL4 expression vectors or ACSL4 siRNA and fostered into DMEM supplemented with 10% FBS and 600 μg/ml HJ11 decoction for 48 h at 37°C, 5% CO_2_, and 95% air.

### Cell counting kit-8 assay

Cell counting kit-8 (CCK-8) assay was performed to determine the cytotoxicity of HJ11 decoction to H9c2 cells. Briefly, 1 × 10^4^ H9c2 cells were cultured in 100 µl of DMEM supplemented with 10% FBS and different concentrations of HJ11 decoction (100, 200, 400, 600, 800, and 1,000 μg/ml) in 96-well plates by incubating them for 48 h at 37°C, 5% CO_2_, and 95% air. Next, CCK-8 solution (10 μl, Biolab Technology Co., Ltd., Beijing, China) was added into each well, and the cell plate was incubated for 2 h at 37°C. The absorbance value of each well was measured using a multi-well microplate reader (Huisong Technology Development Co., Ltd., Shenzhen, China). Cell viability was calculated as follows: (absorbance value of the experimental group/absorbance value of the control group) × 100%. Moreover, the viability of the OGD/R-induced H9c2 cells, OGD/R + HJ11 decoction (100, 200, 400, 600, and 800 μg/ml)–treated H9c2 cells, OGD/R + ACSL4 siRNA–treated H9c2 cells, and OGD/R + pcDNA3.1-ACSL4 vectors + HJ11 decoction (600 μg/ml)–treated H9c2 cells was also measured through the CCK-8 assay. The detection procedure was implemented as described above. In this experiment, untreated H9c2 cells were considered as control cells.

### Detection of iron concentration, reactive oxygen species production, and lipid peroxidation

The blood samples collected from the rats were centrifuged for 10 min at 1,000 × *g* and 4°C to harvest the supernatant. Myocardial tissues in the I/R region of rats were homogenized on ice. The homogenate was then centrifuged for 10 min at 1,000 × *g* and 4°C, and the supernatant was collected. The H9c2 cells of each group were fostered for 48 h in a relevant medium. The cells were collected and lysed for 30 min in the cell lysate (Beyotime, Shanghai, China) on ice, followed by centrifugation for 10 min at 1,000 × *g* and 4°C. The supernatant was then collected. The iron concentration in the supernatant samples was quantified using the Iron Assay Kit (ab83366; Abcam, Cambridge, United Kingdom). Furthermore, the levels of ROS, malondialdehyde (MDA), superoxide dismutase (SOD), and glutathione (GSH) in the supernatant samples of myocardial tissues and H9c2 cells were assessed using the ROS Assay Kit (KA3842; AmyJet Scientific, Wuhan, China), MDA Assay Kit (ab238537; Abcam, Cambridge, United Kingdom), SOD Assay Kit (ab65354; Abcam, Cambridge, United Kingdom), and GSH Assay Kit (ab112132; Abcam, Cambridge, United Kingdom). The detection procedure was followed strictly according to the manufacturers’ instructions.

### Dichloro-dihydro-fluorescein diacetate staining

H9c2 cells were cultured in 6-well plates (1 × 10^6^ cells and 1-ml medium per well) at 37°C, 5% CO_2_, and 95% air in a relevant medium. After 48 h of stimulation, H9c2 cells were washed thrice with PBS. Dichloro-dihydro-fluorescein diacetate (DCFH-DA) solution (1 ml, 10 μM; Beyotime, Shanghai, China) was then added into each well, and the plates were incubated for 20 min in the dark. The residual liquid in each well was removed, and the DCFH-DA-treated H9c2 cells were washed thrice with PBS. DCFH-DA staining was observed under a fluorescence microscope (Olympus, Tokyo, Japan). Green fluorescence was regarded as ROS-positive staining, and the fluorescence intensity was determined using Image-Pro Plus 6.0 software (Media Cybernetics, Inc., Rockville, MD, United States).

### Western blotting

The expression of ferroptosis-related proteins was evaluated by western blotting. The myocardial tissues from the I/R region of rats were homogenized with cell lysates on ice. After centrifugation (1,000 × *g*, 10 min, at 4°C), the supernatant was harvested. H9c2 cells were collected after 48 h of culture in a relevant medium, following which they were lysed for 30 min on ice. The supernatant was collected by centrifugation. The total protein concentration in each supernatant sample was measured using the BCA Kit (Beyotime, Shanghai, China). For each supernatant sample, 60 µg of protein was accumulated for protein separation through sodium dodecyl sulfate–polyacrylamide gel electrophoresis. Afterward, the proteins were blotted onto polyvinylidene fluoride membranes. The blotted proteins were blocked by incubating the membranes with 5% skimmed milk for 1 h at room temperature. The following primary antibodies were used to probe the proteins for 12 h at 4°C: rabbit anti-glutathione peroxidase 4 (GPX4; 1:1000, abs115853; Absin Biotechnology, Shanghai, China), rabbit anti-ferritin heavy polypeptide 1 (FTH1; 1:1000, A-AF1446a; AmyJet Scientific, Wuhan, China), rabbit anti-cyclooxygenase-2 (COX2; 1:1000, ab52237; Abcam, Cambridge, United Kingdom), rabbit anti-ACSL4 (1:1000, K004812P; Solarbio, Beijing, China), and rabbit anti-glyceraldehyde-3-phosphate dehydrogenase (GAPDH; 1:1000, ab70699; Abcam, Cambridge, United Kingdom). The membranes were then washed with Tris-buffered saline containing 0.1% Tween 20 to remove the unbound primary antibodies. The membranes were treated with horseradish peroxidase–conjugated goat antirabbit secondary antibody (1:3000, ab6721; Abcam, Cambridge, United Kingdom) and incubated for 2 h at room temperature. The Enhanced Chemiluminescence Kit (Absin Biotechnology, Shanghai, China) was used for developing specific protein blots in accordance with the manufacturer’s instructions. The protein blots were measured using ImageJ software (National Institutes of Health, Bethesda, MD, United States). The relative expression of proteins was normalized to GAPDH.

### Adenosine triphosphate and mitochondrial DNA detection

The level of adenosine triphosphate (ATP) in the myocardial tissues and H9c2 cells was measured. Tissues and cells were lysed on ice, and the lysate was centrifuged for 5 min at 12,000 × *g* and 4°C to harvest the supernatant. The level of ATP in the supernatant was measured using a commercial ATP assay kit (KA1661; AmyJet Scientific, Wuhan, China) according to the manufacturer’s instructions (Li et al., 2020a).

Additionally, the mitochondrial DNA (mtDNA) level in the myocardial tissues and H9c2 cells was detected as previously reported. Briefly, total DNA in the myocardial tissues and H9c2 cells was extracted using the Puregene DNA isolation kit (Gentra Systems, Minneapolis, MN, United States) in line with the manufacturer’s instructions. The relative content of mtDNA was quantified using qRT-PCR, wherein the mitochondrial gene was detected with GAPDH as the control (Li et al., 2021a).

### Transmission electron microscope

The mitochondrial damage in the myocardial tissues from the I/R region was observed by transmission electron microscopy (TEM). The tissues were fixed in 2.5% pre-cooled glutaraldehyde solution (Solarbio, Beijing, China) for 24 h at 4°C, followed by incubation with 1% osmic acid (Zhenzhun Biotechnology Co., Ltd., Shanghai, China) for 2 h at 4°C. The myocardial tissues were dehydrated in gradient alcohol with a series of ascending concentrations, following which they were embedded into epoxy resin and sectioned at a thickness of 50 nm. The sections were stained with uranyl acetate (Yubo Biotechnology Co., Ltd., Shanghai, China) and lead citrate (Zhongjing Technology Co., Ltd., Beijing, China) and were observed under the JEM-1400 Plus TEM (JEOL, Tokyo, Japan) and photographed.

### qRT-PCR assay

The transfected H9c2 cells were collected, and qRT-PCR assay was performed to evaluate the transfection efficiency. Briefly, H9c2 cells were treated with the TRIzol reagent (Thermo Fisher Scientific, Waltham, MA, United States) to extract total RNA. The total RNA sample (5 µl) was reverse-transcribed into cDNA using the PrimeScript™ RT reagent kit with gDNA Eraser (Takara Bio Inc., Kusatsu, Japan), in line with the manufacturer’s instructions. The PCR assay was performed using the ABI 7500 Fast RT-qPCR system (Applied Biosystems, Foster City, CA, United States) with the SYBR^®^ Premix Ex Taq™ II Kit (Takara, Tokyo, Japan) according to the following parameters: 95°C for 5 min, 40 cycles of (95°C for 30 s, 58°C for 30 s, and 72°C for 30 s), and then 72°C for 5 min. The following primers were used: ACSL4: sense, 5′-CGG​TTC​CTT​TTT​GCG​AGC​TT-3′, antisense, 5′-AAA​GTA​CGC​AAA​TGT​CCT​CTT​TT-3′. GAPDH: sense, 5′-CCA​CTA​GGC​GCT​CAC​TGT​TCT-3′, antisense, 5′-GCA​TCG​CCC​CAC​TTG​ATT​TT-3′. The relative expression of ACSL4 mRNA was determined using the 2^−ΔΔCt^ method, with GAPDH as the control.

### Statistical analysis

In this study, cell experiments were biological replicates. All data were presented as mean ± standard deviation. Between-group difference comparison was analyzed using the two-tailed unpaired Student t-test. One-way analysis of variance and Tukey’s post hoc test were used for assessing the difference comparison between more than two groups. GraphPad Prism 6 software (GraphPad Software, San Diego, CA, United States) was used for the construction of statistical graphs and for the statistical analysis of data. *p* < 0.05 indicated a statistically significant difference.

## Results

### HJ11 decoction improved cardiac function in rats with myocardial I/R injury

A high dose (18 g/kg/d) of HJ11 decoction was administered to normal rats to test baseline toxicity. As displayed in Supplementary Figure S1, after treatment with high-dose HJ11 decoction, the rats had normal ventricular motion, with no damage in the tissues of vital organs, including heart, liver, spleen, lung, and kidney. Thus, a high dose of HJ11 decoction was nontoxic to rats.

**FIGURE 1:**
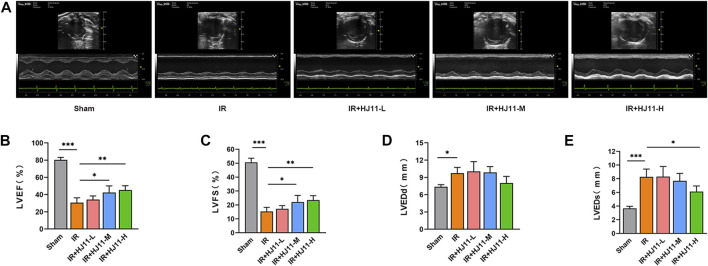
Effect of HJ11 decoction on the cardiac function of rats with myocardial I/R injury. **(A)** Echocardiographic analysis of rats indicating that HJ11 decoction improved the left ventricular anterior wall motion of rats with myocardial ischemia-reperfusion (I/R) injury. **(B–E)** Echocardiographic analysis of rats showing that HJ11 decoction increased left ventricular ejection fraction (LVEF), left ventricular fractional shortening (LVFS), and left ventricular end-systolic diameter (LVEDs; rather than left ventricular end-diastolic diameter [LVEDd]) of rats with myocardial I/R injury. *p < 0.05. **p < 0.01. ***p < 0.001. Experiments were repeated independently, and data are shown as mean ± standard deviation. N = 6 rats for each condition.

After 4-week administration of HJ11 decoction, the cardiac function of rats with myocardial I/R injury was monitored by echocardiography. As displayed in Figure 1A, rats of the Sham group showed normal ventricular motion. However, when compared with the Sham group, rats of the I/R group had markedly weakened left ventricular anterior wall motion. Relative to the I/R group, low-dose HJ11 decoction treatment to the I/R + HJ11-L group showed an apparent improvement in the ventricular wall motion of rats with I/R injury. Importantly, the administration of HJ11 decoction at medium and high doses (I/R + HJ11-M group and I/R + HJ11-H group) greatly improved ventricular wall motion in rats with I/R injury, when compared with that in rats of the I/R group.

Additionally, the LVEF, LVFS, LVEDd, and LVEDs of rats in all the groups were scrutinized, and the results are presented in Figures 1B–E. Rats of the I/R group had reduced LVEF and LVFS as well as elevated LVEDd and LVEDs when compared with rats of the Sham group (*p* < 0.05 and *p* < 0.001). Intriguingly, HJ11 decoction at medium and high doses (I/R + HJ11-M group and I/R + HJ11-H group) significantly increased the LVEF and LVFS in rats with I/R relative to the LVEF and LVFS in the I/R group (*p* < 0.05). Moreover, when compared with the I/R group, HJ11 decoction treatment at a high dose (I/R + HJ11-H group) showed an evident reduction in the LVEDs in rats with I/R (*p* < 0.05). However, HJ11 decoction treatment did not show an apparent change in the LVEDd in rats with I/R when compared with that in the I/R group.

### HJ11 decoction alleviated myocardial injury in rats with myocardial I/R

**FIGURE 2:**
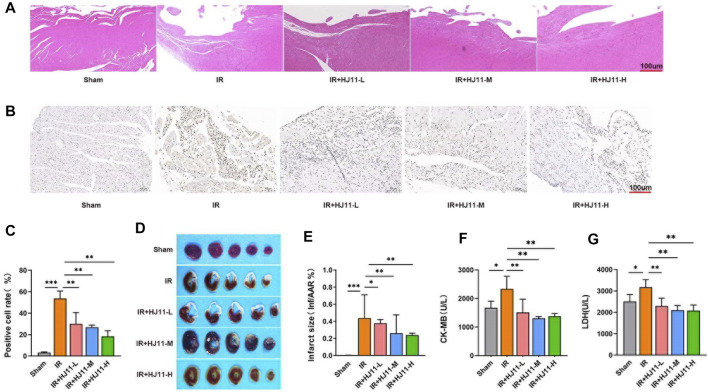
Effect of HJ11 decoction on myocardial I/R injury in the rat model. **(A)** Hematoxylin-eosin staining showing that HJ11 decoction reduced myocardial tissue injury in rats with myocardial ischemia-reperfusion (I/R) injury. Scale bar: 100 µm. **(B,C)** Terminal deoxynucleotidyl transferase dUTP nick end labeling (TUNEL) staining and statistical analysis of TUNEL-positive cells indicated that HJ11 decoction alleviated apoptosis in the myocardial tissues of rats with myocardial I/R injury. Scale bar: 100 µm. **(D,E)** 2,3,5-Triphenyltetrazolium chloride (TTC) staining of myocardial tissues and statistical analysis suggested that HJ11 decoction reduced the infarct size of rats with myocardial I/R injury. **(F,G)** HJ11 decoction decreased the levels of creatine kinase–MB (CK-MB) and lactate dehydrogenase (LDH) in the serum of rats. *p < 0.05. **p < 0.01. ***p < 0.001. Data are shown as mean ± standard deviation. N = 3–6 rats for each condition.

Myocardial injury in rats was evaluated through HE staining. The results showed that rats of the Sham group had neatly arranged myocardial cells, evenly colored myocardial fibers, clear and regular horizontal stripes of myocardial cells, and no evident inflammatory cell infiltration. However, rats of the I/R group had severely damaged myocardial tissue structure, disorganized cardiomyocytes, damaged cardiomyocyte structure, and severe inflammatory cell infiltration. Relative to the I/R group, rats of the HJ11 decoction treatment groups (I/R + HJ11-L group, I/R + HJ11-M group, and I/R + HJ11-H group) showed alleviation of the damage to the myocardial tissue structure and cardiomyocytes and attenuation of inflammatory cell infiltration. Among the I/R + HJ11-L, I/R + HJ11-M, and I/R + HJ11-H groups, rats of the I/R + HJ11-H group had the greatest alleviation of myocardial injury (Figure 2A).

TUNEL assay was performed to detect the apoptosis of cardiomyocytes in the myocardial tissues of rats. Rats of the I/R group showed a higher increase in the TUNEL-positive cell rate than those of the Sham group (*p* < 0.001). Intriguingly, compared with the I/R group, a much higher reduction in the TUNEL-positive cell rate was observed in rats of the I/R + HJ11-L, I/R + HJ11-M, and I/R + HJ11-H groups (*p* < 0.01; Figures 2B,C).

TTC staining was performed to detect the infarct size. Rats of the I/R group had prominently larger infarct size than those of the Sham group (*p* < 0.001). Rats of the HJ11 decoction treatment groups (I/R + HJ11-L, I/R + HJ11-M, and I/R + HJ11-H groups) showed a marked decline in the infarct size than those of the I/R group (*p* < 0.05 and *p* < 0.01; Figures 2D,E).

The levels of CK-MB and LDH in the myocardial tissues of rats were measured using commercial kits. The results showed that rats of the I/R group had much-increased levels of CK-MB and LDH when compared with those of the Sham group (*p* < 0.05). However, when compared with the I/R group, rats of the I/R + HJ11-L, I/R + HJ11-M, and I/R + HJ11-H groups had reduced levels of CK-MB and LDH (*p* < 0.01; Figures 2F,G). Thus, these results suggest that HJ11 decoction treatment could attenuate myocardial injury in rats with myocardial I/R.

### HJ11 decoction suppressed myocardial I/R injury–induced oxidative stress and iron accumulation in rats

**FIGURE 3:**
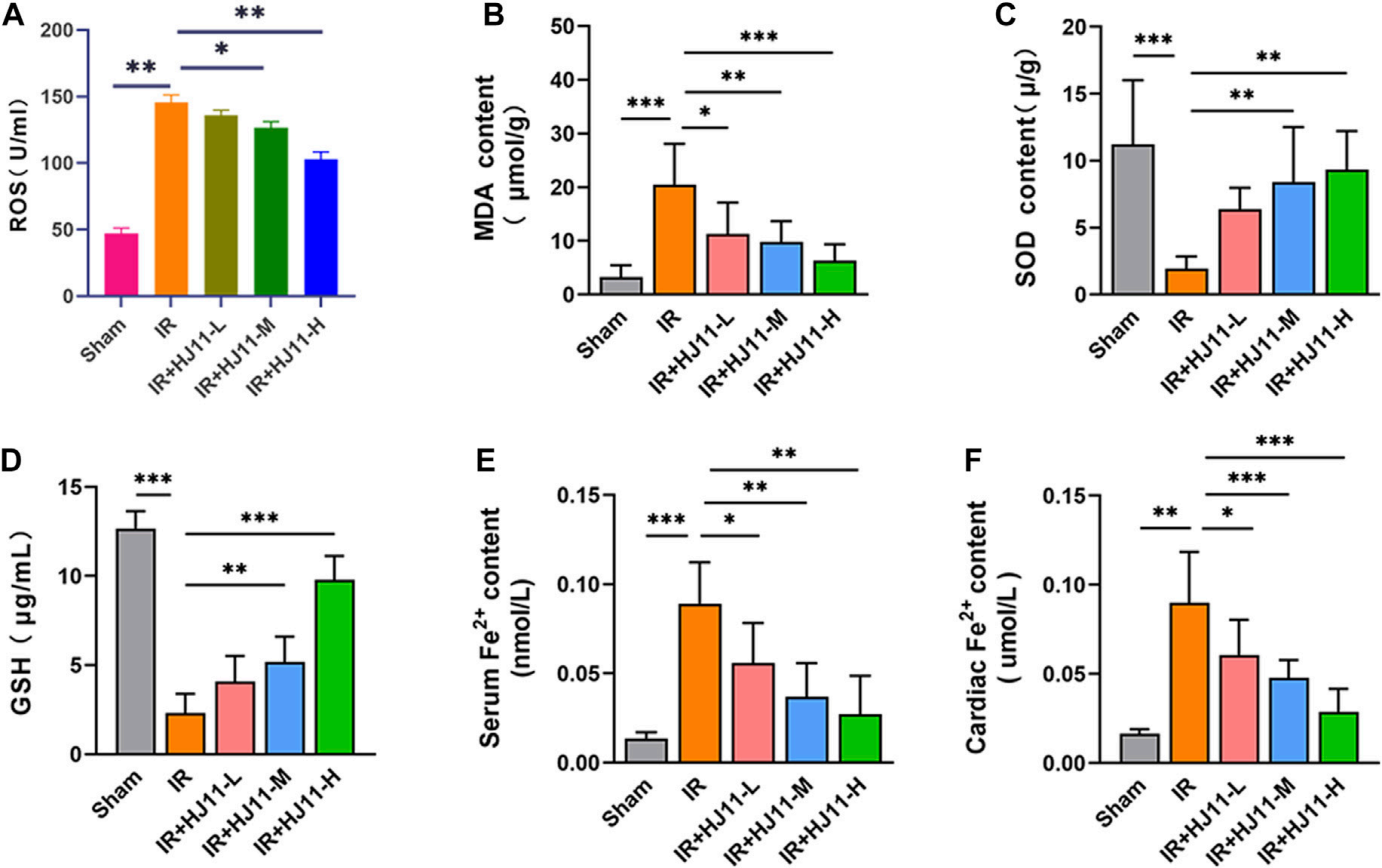
Effect of HJ11 decoction on oxidative stress and iron ion levels in rats with myocardial I/R injury. **(A–D)** HJ11 decoction decreased the levels of reactive oxygen species and malondialdehyde but increased the levels of superoxide dismutase and glutathione in the myocardial tissues of rats with myocardial ischemia-reperfusion (I/R) injury. **(E,F)** HJ11 decoction reduced the level of iron ion accumulation in the serum and myocardial tissues of rats with myocardial I/R injury. *p < 0.05. **p < 0.01. ***p < 0.001. Data are shown as mean ± standard deviation. N = 3–6 rats for each condition.

The levels of ROS, MDA, SOD, and GSH in the myocardial tissues of rats were quantified to evaluate oxidative stress. Rats of the I/R group exhibited higher ROS and MDA levels as well as lower SOD and GSG levels in the myocardial tissues than rats of the Sham group (*p* < 0.01 and *p* < 0.001). Interestingly, HJ11 decoction at medium and high doses (I/R + HJ11-M group and I/R + HJ11-H group) remarkably reduced ROS and MDA levels and increased SOD and GSH levels in the myocardial tissues of rats relative to those in the I/R group (*p* < 0.05, *p* < 0.01; Figures 3A–D).

Next, the iron ion level in the serum and myocardial tissues of rats was measured, and the results are shown in Figures 3E,F. A higher iron ion level in the serum and myocardial tissues was observed in rats of the I/R group relative to that in the Sham group (*p* < 0.01 and *p* < 0.001). When compared with the I/R group, rats of the three HJ11 decoction treatment groups (I/R + HJ11-L group, I/R + HJ11-M group, and I/R + HJ11-H group) showed a remarkably reduced iron ion level in the serum and myocardial tissues (*p* < 0.05 and *p* < 0.001). Hence, these results suggested that HJ11 decoction inhibited myocardial I/R injury–induced oxidative stress and iron ion accumulation in rats.

### HJ11 decoction reduced myocardial I/R injury–induced iron accumulation in the myocardial tissues of rats

**FIGURE 4:**
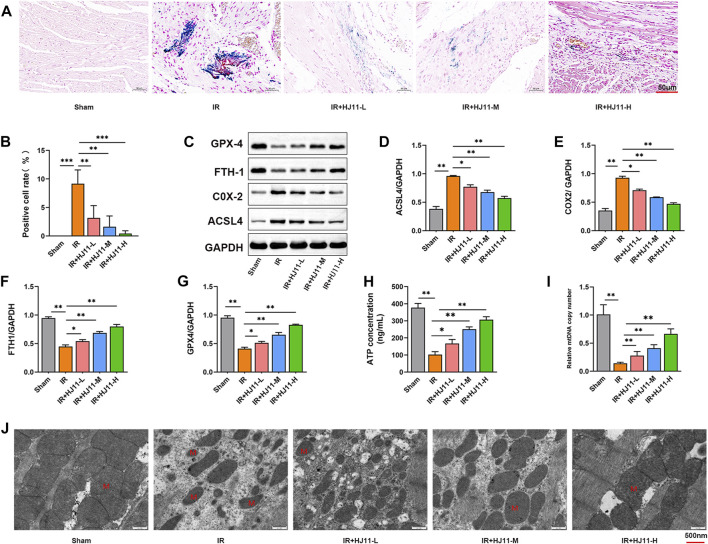
Effects of HJ11 decoction on iron ion accumulation and mitochondrial damage in rats with myocardial I/R injury. **(A,B)** Representative images of Prussian blue staining and statistical analysis illustrating that HJ11 decoction attenuated iron accumulation in the myocardial tissues of rats with myocardial ischemia-reperfusion (I/R) injury. Scale bar: 50 µm. **(C–G)** Representative western blots for ferroptosis-associated proteins in the myocardial tissues of rats. Statistical analysis of western blots indicated that HJ11 decoction suppressed the expression of ACSL4 and COX2 proteins but promoted the expression of FTH1 and GPX4 proteins in the myocardial tissues of rats with myocardial I/R injury. **(H,I)** HJ11 decoction enhanced adenosine triphosphate (ATP) and mitochondrial DNA (mtDNA) levels in the myocardial tissues of rats with myocardial I/R injury. **(J)** Representative transmission electron microscopy images of mitochondria in the myocardial tissues of rats, indicating that HJ11 decoction alleviated mitochondrial damage in the myocardial tissues of rats with myocardial I/R injury. Scale bar: 500 nm *p < 0.05. **p < 0.01. ***p < 0.001. Data are shown as mean ± standard deviation. N = 3 rats for each condition.

Iron accumulation in the myocardial tissues of rats was assessed through Prussian blue staining. Prussian blue staining was barely observed in the myocardial tissues of rats in the Sham group when compared with that in rats of the I/R group, wherein remarkably intense staining was noted in the myocardial tissues (*p* < 0.001). When compared with the I/R group, rats of the three HJ11 decoction treatment groups (I/R + HJ11-L, I/R + HJ11-M, and I/R + HJ11-H groups) displayed distinctly attenuated Prussian blue staining in the myocardial tissues (*p* < 0.01 and *p* < 0.001; Figures 4A,B).

Western blotting was performed to study the expression of ferroptosis-associated proteins, including ACSL4, COX2, FTH1, and GPX4 in the myocardial tissues of rats. When compared with the Sham group, rats in the I/R group had elevated expression levels of ACSL4 and COX2 proteins as well as reduced expression levels of FTH1 and GPX4 proteins in the myocardial tissues (*p* < 0.01). However, rats of the three HJ11 decoction treatment groups (I/R + HJ11-L, I/R + HJ11-M, and I/R + HJ11-H groups) had lower expression levels of ACSL4 and COX2 proteins and higher expression levels of FTH1 and GPX4 proteins than those of the I/R group (*p* < 0.05 and *p* < 0.01; Figures 4C–G).

The effect of HJ11 decoction on metabolism-related indicators, including ATP and mtDNA levels was evaluated. Rats of the I/R group exhibited lower ATP and mtDNA levels in the myocardial tissues than rats of the Sham group (*p* < 0.01). Intriguingly, much elevated ATP and mtDNA levels were observed in the three HJ11 decoction treatment groups (I/R + HJ11-L, I/R + HJ11-M, and I/R + HJ11-H groups) when compared with those in the I/R group (*p* < 0.05 and *p* < 0.01; Figures 4H,I).

The mitochondrial structure in the myocardial tissues of rats was observed by TEM. Rats of the Sham group had a regular mitochondrial structure. However, a severely distorted and disrupted mitochondrial structure was observed in the myocardial tissues of rats in the I/R group. HJ11 decoction treatment (I/R + HJ11-L, I/R + HJ11-M, and I/R + HJ11-H groups) mitigated the damage to the mitochondrial structure to varying degrees when compared with that in the I/R group (Figure 4J). Therefore, HJ11 decoction inhibited iron accumulation and mitochondrial damage in the myocardial tissues of rats with I/R.

### HJ11 decoction protected myocardial H9c2 cells from injury due to oxygen glucose deprivation/reperfusion-triggered oxidative stress

**FIGURE 5:**
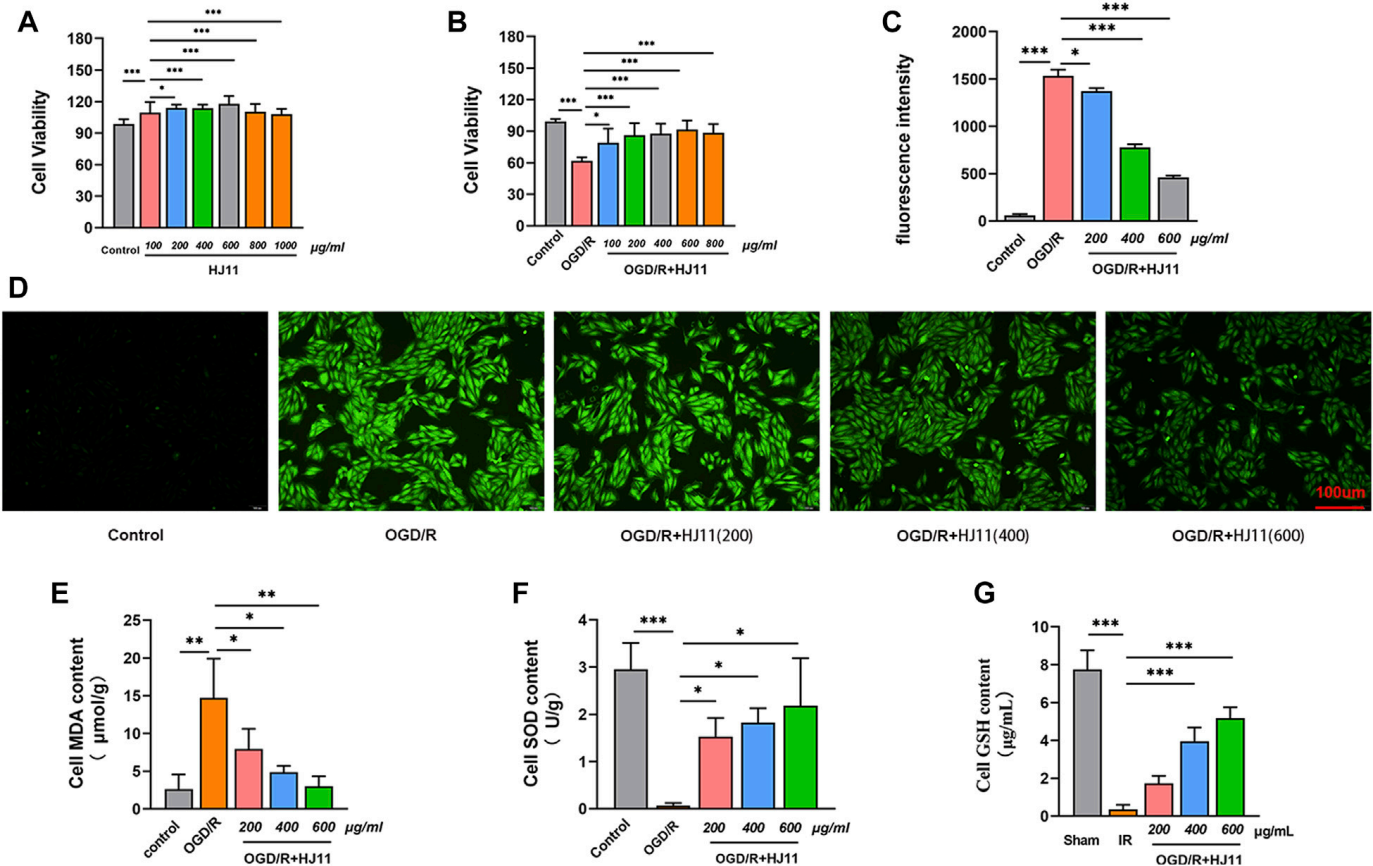
Effects of HJ11 decoction on OGD/R-triggered injury in H9c2 cells. **(A,B)** Cytotoxicity assay with HJ11 decoction application to normal H9c2 cells and oxygen-glucose deprivation-reperfusion (OGD/R)–induced H9c2 cells suggesting that HJ11 decoction was nontoxic to normal H9c2 cells at doses of 100–1000 μg/ml and to OGD/R-induced H9c2 cells at doses of 100–800 μg/ml. **(C,D)** Dichloro-dihydro-fluorescein diacetate staining and statistical analysis showing that HJ11 decoction reduced the level of reactive oxygen species in OGD/R-induced H9c2 cells. Scale bar: 100 µm. **(E–G)** HJ11 decoction reduced the level of malondialdehyde but elevated the levels of superoxide dismutase and glutathione in the OGD/R-induced H9c2 cells. *p < 0.05. **p < 0.01. ***p < 0.001. Data are shown as mean ± standard deviation. N = 3–4 for each condition.

To verify the cytotoxicity effect of HJ11 decoction to H9c2 cells, CCK-8 assay was performed on H9c2 cells treated with different concentrations of HJ11 decoction. HJ11 decoction at doses of 100, 200, 400, 600, 800, and 1000 μg/ml was nontoxic to H9c2 cells (Figure 5A). Simultaneously, the viability of H9c2 cells started to decline with HJ11 decoction at doses of 800 and 1,000 μg/ml. Therefore, HJ11 decoction at a dose range of 100–800 μg/ml was used for treating the OGD/R-induced H9c2 cells.

HJ11 decoction was used at a dose range of 100–800 μg/ml to treat the OGD/R-induced H9c2 cells to determine the appropriate HJ11 decoction dose that does not destroy cell viability. The viability of H9c2 cells in the OGD/R group was much lower than that in the control group (*p* < 0.001). When compared with the OGD/R group, HJ11 decoction treatment (at doses of 100, 200, 400, 600, and 800 μg/ml) greatly improved the viability of the OGD/R-induced H9c2 cells (*p* < 0.05 and *p* < 0.001). Moreover, an increasing trend was noted in the improvement effect of HJ11 decoction on the viability of H9c2 cells from doses of 100–600 μg/ml. However, HJ11 decoction at a dose of 800 μg/ml reduced the viability of H9c2 cells when compared with the dose of 600 μg/ml (Figure 5B). Therefore, in subsequent studies, HJ11 decoction was used at doses of 200, 400, and 600 μg/ml to treat H9c2 cells.

Subsequently, the influence of HJ11 decoction on the ROS level in H9c2 cells was evaluated through DCFH-DA staining, and the results are presented in Figures 5C,D. The ROS fluorescence intensity was enhanced in H9c2 cells of the OGD/R group relative to that in the control group (*p* < 0.001). HJ11 decoction treatment at doses of 200, 400, and 600 μg/ml (OGD/R + HJ11–200 μg/ml group, OGD/R + HJ11–400 μg/ml group, and OGD/R + HJ11–600 μg/ml group) prominently attenuated ROS fluorescence intensity relative to that in the OGD/R group (*p* < 0.05 and *p* < 0.001).

Additionally, the MDA, SOD, and GSH levels in H9c2 cells of each group were tested using commercial kits, and the results are shown in Figures 5E–G. When compared with the control group, a higher level of MDA and lower levels of SOD and GSH were noted in H9c2 cells of the OGD/R group (*p* < 0.01 and *p* < 0.001). The MDA level was distinctly reduced and the SOD level was elevated in H9c2 cells of the OGD/R + HJ11–200 μg/ml group, OGD/R + HJ11–400 μg/ml group, and OGD/R + HJ11–600 μg/ml group when compared with those in the OGD/R group (*p* < 0.05 and *p* < 0.01). Furthermore, the GSH level was increased in H9c2 cells of the OGD/R + HJ11–400 μg/ml group and the OGD/R + HJ11–600 μg/ml group relative to that in the OGD/R group (*p* < 0.001). All these findings suggest that HJ11 decoction could confer protection to H9c2 cells from injury due to OGD/R-triggered oxidative stress.

### HJ11 decoction weakened oxygen glucose deprivation/reperfusion-triggered iron accumulation and mitochondrial damage in H9c2 cells

**FIGURE 6:**
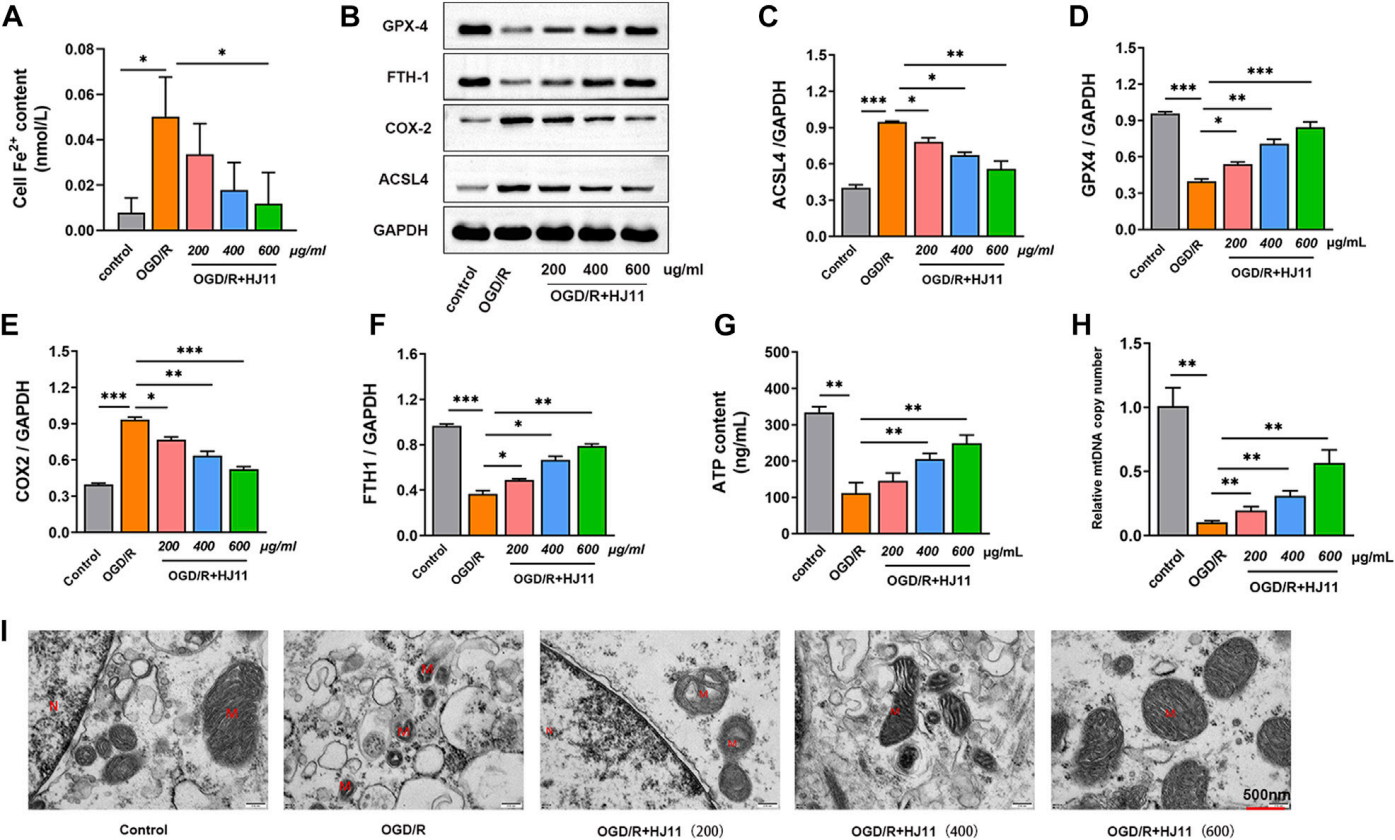
Effects of HJ11 decoction on iron accumulation and mitochondrial damage in H9c2 cells. **(A)** HJ11 decoction decreased the level of iron accumulation in oxygen glucose deprivation-reperfusion (OGD/R)–induced H9c2 cells. **(B–F)** Representative western blot images for ferroptosis-associated proteins in H9c2 cells. Statistical analysis of western blots illustrating that HJ11 decoction inhibited the expression of ACSL4 and COX2 proteins but promoted the expression of FTH1 and GPX4 proteins in the OGD/R-induced H9c2 cells. **(G,H)** HJ11 decoction increased the levels of adenosine triphosphate (ATP) and mitochondrial DNA (mtDNA) in the OGD/R-induced H9c2 cells. **(I)** Representative transmission electron microscopy images of mitochondria, indicating that HJ11 decoction alleviated mitochondrial damage in the OGD/R-induced H9c2 cells. Scale bar: 500 nm *p < 0.05. **p < 0.01. ***p < 0.001. Data are shown as mean ± standard deviation. N = 3–4 for each condition.

This study detected the effect of HJ11 decoction on OGD/R-triggered iron accumulation in H9c2 cells. The increase in the iron level was higher in H9c2 cells of the OGD/R group than in those of the control group (*p* < 0.05). A high dose (600 μg/ml) of HJ11 decoction treatment evidently reduced the iron level in the OGD/R-induced H9c2 cells when compared with that in the OGD/R group (*p* < 0.05). However, HJ11 decoction at doses of 200 and 400 μg/ml had no influence on the iron level when compared with that in the OGD/R group (Figure 6A).

Western blotting results indicated the downregulated expression of FTH1 and GPX4 proteins and the upregulated expression of ACSL4 and COX2 proteins in H9c2 cells of the OGD/R group when compared with that in the control group (*p* < 0.001). Relative to the OGD/R group, H9c2 cells of the three HJ11 decoction treatment groups (OGD/R + HJ11–200 μg/ml group, OGD/R + HJ11–400 μg/ml group, and OGD/R + HJ11–600 μg/ml group) showed increased levels of FTH1 and GPX4 proteins and decreased levels of ACSL4 and COX2 proteins (*p* < 0.05, *p* < 0.01, and *p* < 0.001; Figures 6B–F).

Moreover, H9c2 cells of the OGD/R group had lower levels of ATP and mtDNA than those of the control group (*p* < 0.01). Relative to the OGD/R group, HJ11 decoction at doses of 400 and 600 μg/ml significantly increased the ATP level, and at doses of 200, 400, and 600 μg/ml showed a higher increase in the mtDNA level in the OGD/R-treated H9c2 cells (Figures 6G,H). According to TEM results, H9c2 cells of the control group had a normal mitochondrial structure, whereas those of the OGD/R group had a damaged mitochondrial structure (such as shrinkage of mitochondria and broken mitochondrial cristae). HJ11 decoction treatment (OGD/R + HJ11–200 μg/ml group, OGD/R + HJ11–400 μg/ml group, and OGD/R + HJ11–600 μg/ml group) showed an apparent improvement in OGD/R-induced mitochondrial damage (Figure 6I). Therefore, these findings suggest that HJ11 decoction weakens OGD/R-triggered iron accumulation and mitochondrial damage in H9c2 cells.

### Overexpression of ACSL4 reversed the protective effect of HJ11 decoction on oxygen glucose deprivation/reperfusion-treated H9c2 cells

**FIGURE 7:**
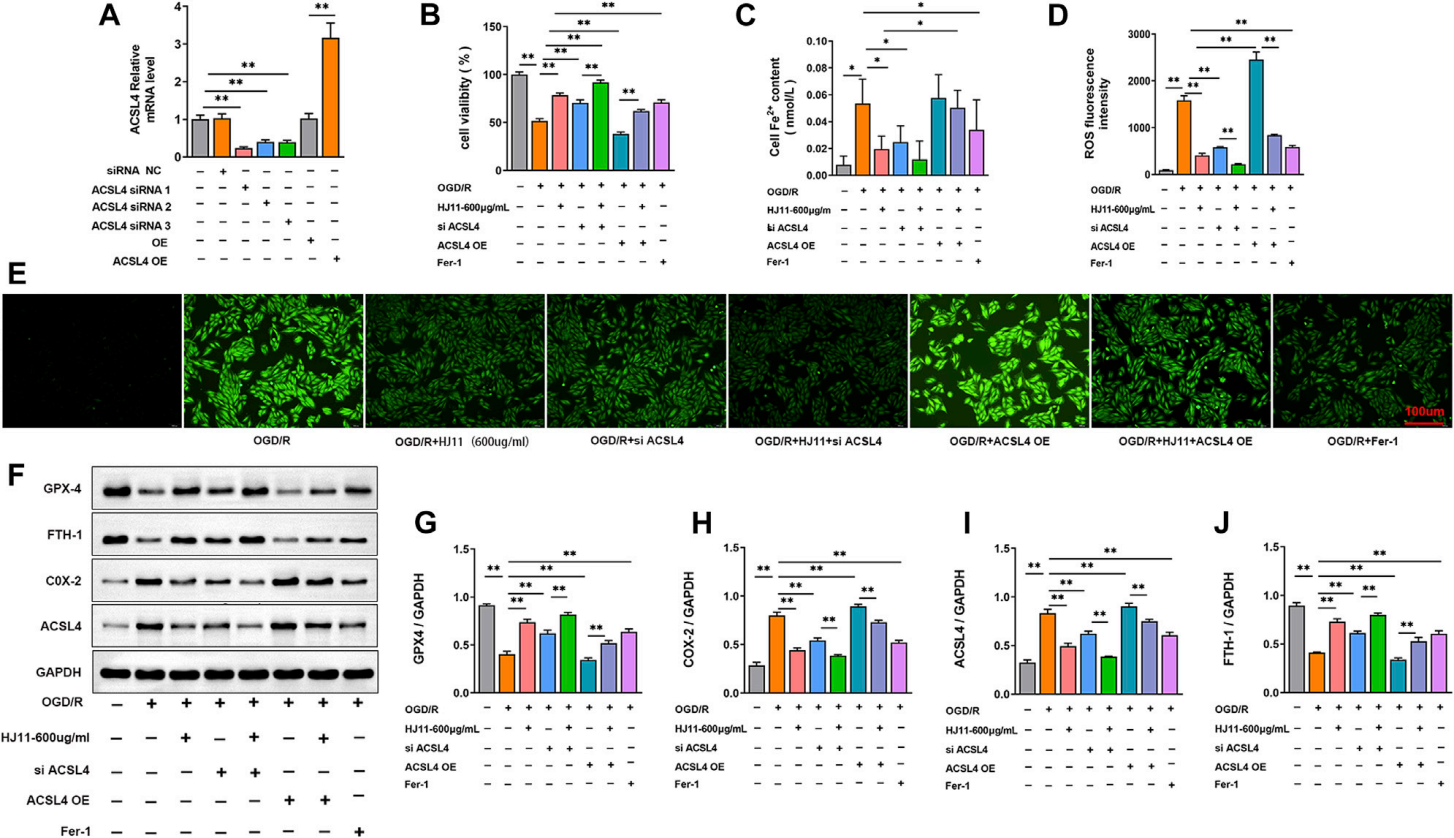
Effects of HJ11 decoction and ACSL4 on OGD/R-triggered injury in H9c2 cells. **(A)** Transfection efficiency detection by quantitative reverse transcription–polymerase chain reaction (qRT-PCR) assay indicating that ACSL4 small interfering RNA (siRNA) and pcDNA3.1-ACSL4 vectors were successfully transfected into H9c2 cells. **(B)** Cell Counting Kit-8 assay showing that ACSL4 overexpression reversed the promoting effect of HJ11 decoction on the viability of oxygen glucose deprivation-reperfusion (OGD/R)–induced H9c2 cells. **(C)** ACSL4 overexpression counteracted the suppressing effect of HJ11 decoction on the iron accumulation level in the OGD/R-induced H9c2 cells. **(D,E)** Dichloro-dihydro-fluorescein diacetate staining and statistical analysis indicating that ACSL4 overexpression abrogated the inhibitory effect of HJ11 decoction on the production of reactive oxygen species in the OGD/R-induced H9c2 cells. Scale bar: 100 µm. **(F–J)** Representative western blot images of ferroptosis-associated proteins in H9c2 cells. Statistical analysis of western blots implying that ACSL4 overexpression reversed the suppressing effect of HJ11 decoction on the expression of ACSL4 and COX2 proteins and the promoting effect of HJ11 decoction on the expression of FTH1 and GPX4 proteins in the OGD/R-induced H9c2 cells. *p < 0.05. **p < 0.01. ***p < 0.001. Data are shown as mean ± standard deviation. N = 3 for each condition.

To verify whether HJ11 decoction exerted a protective effect against OGD/R-triggered injury in H9c2 cells by regulating ACSL4, transfection of ACSL4 siRNA and pcDNA3.1-ACSL4 vectors was performed on H9c2 cells. The transfection efficiency was scrutinized through qRT-PCR. As shown in Figure 7A, ACSL4 mRNA expression was much reduced in H9c2 cells of the ACSL4 siRNA-1, -2, and -3 groups when compared with that in the siRNA NC group (*p* < 0.01). Conversely, H9c2 cells of the ACSL4 OE group showed much higher expression of ACSL4 mRNA than those of the OE group (*p* < 0.01). Thus, ACSL4 siRNA and pcDNA3.1-ACSL4 vectors were successfully transfected into H9c2 cells. Among the ACSL4 siRNA-1, -2, and -3 groups, H9c2 cells of the siRNA-1 group demonstrated the lowest expression of ACSL4 mRNA. Thus, ACSL4 siRNA-1 was applied to transfect the OGD/R-induced H9c2 cells in the present research.

CCK-8 assay was performed to study the viability of H9c2 cells in each group, and the results are presented in Figure 7B. The viability was attenuated in the H9c2 cells of the OGD/R group when compared with that of the H9c2 cells in the control group (*p* < 0.01). However, relative to the OGD/R group, H9c2 cells of the OGD/R + HJ11–600 μg/ml group, the cells of the OGD/R + siACSL4 group, OGD/R + HJ11 + siACSL4 group, and OGD/R + Fer-1 group showed intensified viability (*p* < 0.01). H9c2 cells of the OGD/R + ACSL4 OE group had reduced viability relative to those of the OGD/R group (*p* < 0.01). When compared with the OGD/R + siACSL4 group, H9c2 cells of the OGD/R + HJ11 + siACSL4 group showed intensified viability (*p* < 0.01). Meanwhile, the viability was much higher in H9c2 cells of the OGD/R + HJ11 + ACSL4 OE group relative to that in the OGD/R + ACSL4 OE group (*p* < 0.01). Additionally, a higher level of iron accumulation was observed in H9c2 cells of the OGD/R group relative to that in the control group (*p* < 0.05). However, a prominently lower level of iron accumulation occurred in H9c2 cells of the OGD/R + HJ11–600 μg/ml group, OGD/R + siACSL4 group, and OGD/R + Fer-1 group relative to that in the OGD/R group (*p* < 0.05). When compared with the OGD/R + HJ11–600 μg/ml group, the iron level showed an increase in H9c2 cells of the OGD/R + HJ11–600 μg/ml + ACSL4 OE group (*p* < 0.05; Figure 7C).

As shown in Figures 7D,E, the ROS level in H9c2 cells was evaluated through DCFH-DA staining. The ROS fluorescence intensity was increased in H9c2 cells of the OGD/R group relative to that in the control group (*p* < 0.01). Conversely, compared with H9c2 cells of the OGD/R group, a lower ROS fluorescence intensity was observed in the OGD/R + HJ11–600 group, OGD/R + siACSL4 group, and OGD/R + Fer-1 group but a higher ROS fluorescence intensity was seen in the OGD/R + ACSL4 OE group (*p* < 0.01). Relative to the OGD/R + ACSL4 OE group, H9c2 cells of the OGD/R group and OGD/R + HJ11–600 + ACSL4 OE group displayed a lower ROS fluorescence intensity (*p* < 0.01). Additionally, in contrast to the OGD/R + siACSL4 group, a reduction in ROS fluorescence intensity was observed in H9c2 cells of the OGD/R + HJ11–600 + siACSL4 group (*p* < 0.01).

Western blotting was performed to determine the expression of ferroptosis-associated proteins in H9c2 cells of each group. Relative to the control group, H9c2 cells of the OGD/R group had increased expression of COX2 and ACSL4 proteins and decreased expression of GPX4 and FTH1 proteins (*p* < 0.01). On the contrary, relative to the OGD/R group, distinctly lower expression of COX2 and ACSL4 proteins and higher expression of GPX4 and FTH1 proteins were noted in H9c2 cells of the OGD/R + HJ11–600 group, OGD/R + siACSL4 group, and OGD/R + Fer-1 group (*p* < 0.01). However, H9c2 cells of the OGD/R + ACSL4 OE group had higher expression of COX2 and ACSL4 proteins and lower expression of GPX4 and FTH1 proteins than the OGD/R group (*p* < 0.01). Relative to the OGD/R + siACSL4 group, H9c2 cells of the OGD/R + HJ11 + siACSL4 group showed lower expression of COX2 and ACSL4 proteins but higher expression of GPX4 and FTH1 proteins (*p* < 0.01). Simultaneously, lower expression of COX2 and ACSL4 proteins and higher expression of GPX4 and FTH1 proteins were observed in H9c2 cells of the OGD/R + HJ11 + ACSL4 OE group when compared with those of the OGD/R + ACSL4 OE group (*p* < 0.01) (Figures 7F–J).

Furthermore, the MDA, SOD, and GSH levels in the H9c2 cells of each group were measured using commercial kits. A prominently higher MDA level and lower GSH levels were noted in the H9c2 cells of the OGD/R group than in those of the control group (*p* < 0.05 and *p* < 0.01). When compared with the OGD/R group, the GSH level increased in H9c2 cells of the OGD/R + HJ11–600 group, OGD/R + siACSL4 group, OGD/R + HJ11–600 + siACSL4 group, and OGD/R + Fer-1 group (*p* < 0.01). Meanwhile, H9c2 cells of the OGD/R + HJ11–600 + ACSL4 OE group showed a higher GSH level than those of the OGD/R + ACSL4 OE group (*p* < 0.01). Furthermore, a reduced MDA level was observed in H9c2 cells of the OGD/R + HJ11–600 group, OGD/R + siACSL4 group, and OGD/R + Fer-1 group (*p* < 0.05). When compared with the OGD/R + HJ11–600 group, H9c2 cells of the OGD/R + HJ11–600 + ACSL4 OE group exhibited an elevated MDA level (*p* < 0.05; Supplementary Figures S2A,B). Additionally, a much lower SOD level was noted in H9c2 cells of the OGD/R group than in those of the control group and OGD/R + HJ11–600 group (*p* < 0.05; Supplementary Figure S2C).

Analysis of the ATP and mtDNA levels showed a higher reduction in H9c2 cells of the OGD/R group than in those of the control group (*p* < 0.01). Conversely, relative to the OGD/R group, H9c2 cells of the OGD/R + HJ11–600 group, OGD/R + siACSL4 group, OGD/R + HJ11–600 + siACSL4 group, and OGD/R + Fer-1 group had higher ATP and mtDNA levels (*p* < 0.01). However, the ATP and mtDNA levels had reduced in H9c2 cells of the OGD/R + ACSL4 OE group when compared with those in the OGD/R group (*p* < 0.01). H9c2 cells of the OGD/R + HJ11–600 + ACSL4 OE group had a lower ATP level than those of the OGD/R + HJ11–600 group (*p* < 0.05). Simultaneously, a higher mtDNA level was seen in the OGD/R + HJ11–600 + siACSL4 group than in the OGD/R + siACSL4 group (*p* < 0.01). Similarly, compared with the OGD/R + ACSL4 OE group, H9c2 cells of the OGD/R + HJ11–600 + ACSL4 OE group exhibited a higher mtDNA level (*p* < 0.01; Supplementary Figures S2D,E). All these findings implied that overexpression of ACSL4 reversed the protective effect of HJ11 decoction against OGD/R-triggered injury in H9c2 cells.

## Discussion

This research explored the effect of HJ11 decoction on myocardial I/R injury. The findings suggested that HJ11 decoction was effective in improving heart function and alleviating myocardial I/R injury. Mechanically, HJ11 decoction might alleviate myocardial I/R injury by inhibiting ACSL4-mediated ferroptosis. In this study, HJ11 decoction was administered to rats through gavage, and this mode of drug administration was the same as that in humans. Therefore, the biological mechanism underlying the alleviation of myocardial I/R injury in rats was found to be the same as that in humans, which provided an important basis for the clinical use of HJ11 decoction in the clinical setting. As previously reported, the active ingredients of traditional Chinese medicine are mostly small-molecule compounds (Li et al., 2020b). Thus, these small-molecule compounds in HJ11 decoction might be the main components that induce biological effects against myocardial I/R injury.

Myocardial I/R injury is regarded as a critical factor for the recovery of cardiac function and is an important factor affecting patient prognosis. The pathophysiology of myocardial I/R injury involves complex mechanisms such as inflammation, mitochondrial dysfunction, microvascular dysfunction, intracellular calcium overload, oxidative stress, cardiomyocyte apoptosis, and metabolic derangements. (Li et al., 2019; Li et al., 2021b; Li et al., 2022). Of these mechanisms, as one of the main forms of cell death, the apoptosis of cardiomyocytes is a fundamental pathogenic factor for myocardial I/R injury (Li et al., 2021c). A study conducted in an animal myocardial I/R injury model has confirmed that blocking cardiomyocyte apoptosis is conducive to attenuating myocardial I/R injury (Li et al., 2017). Interestingly, this research demonstrated that HJ11 decoction could mitigate the apoptosis of cardiomyocytes in the myocardial I/R injury rat model. CK-MB and LDH are sensitive factors for the evaluation of myocardial I/R injury (Liu et al., 2022). Aberrant expression of CK-MB and LDH can suppress the antioxidant potential of cardiomyocytes (Liu et al., 2020). The findings of this study suggest that HJ11 decoction treatment reduced the levels of CK-MB and LDH in the myocardial tissues. Thus, HJ11 decoction might alleviate myocardial I/R injury by enhancing the antioxidant potential of cardiomyocytes by repressing the expression of CK-MB and LDH.

Ferroptosis is a major factor in inducing myocardial I/R injury, and the level of iron accumulation in cardiomyocytes is regarded as a key prognostic factor for myocardial I/R injury (Lu et al., 2021; Lv et al., 2021). The biochemical signature of ferroptosis is the excessive accumulation of iron and ROS-mediated lipid peroxidation (Ma et al., 2020). Uncontrolled accumulation of iron ions can drive the overproduction of ROS and upregulation of lipid peroxidation, thereby resulting in enormous toxicity to cardiomyocytes (Park et al., 2020; Lv et al., 2021). However, the intensified oxidative stress can further expand the myocardial infarct area (Samidurai et al., 2021). In this research, HJ11 decoction showed an evident reduction in the level of iron accumulation in the serum and myocardial tissues and reduced the size of the myocardial infarct area. Meanwhile, it decreased the levels of ROS and MDA but increased the levels of SOD and GSH in the myocardial tissues of rats with myocardial I/R. ROS accumulation can disrupt calcium homeostasis, thereby attenuating cardiomyocyte viability and, finally, resulting in death (Beom et al., 2021). MDA is one of the major products of lipid peroxidation, and SOD is the key enzyme system for scavenging oxygen free radicals. Therefore, MDA and SOD are important factors for evaluating the degree of lipid peroxidation (Shan et al., 2021). GSH is a pivotal antioxidant that confers protection against lipid peroxidation and ROS-induced cell damage by neutralizing ROS (Sun et al., 2022). Thus, HJ11 decoction might confer protection to the myocardial tissue against I/R injury by inhibiting ferroptosis-mediated lipid peroxidation. Intriguingly, a high dose of HJ11 decoction was found to rescue antioxidants and restore the iron ion level to almost the normal level in rats of the Sham group. However, when treated with a high dose of HJ11 decoction, the ROS level remained higher than that in rats of the Sham group. As previously reported, cellular energy metabolism and oxygen consumption are related to ROS production, and a reduction in the metabolic rate can suppress ROS production (Tian et al., 2020). In this study, a high dose of HJ11 decoction treatment enhanced the metabolic rate of rats with myocardial I/R injury, as evidenced by the increased ATP level. Thus, HJ11 decoction might intensify the metabolic rate of rats with myocardial I/R injury, which further resulted in a higher ROS level than that in rats of the Sham group.

Mitochondrial damage is the main morphological characteristic feature of ferroptosis, such as shrinkage of the mitochondria and broken mitochondrial cristae (Cui et al., 2021). The mitochondrion is a major organelle involved in ATP production to provide the necessary cellular energy. The damaged mitochondria can not only stimulate the release of apoptotic factors but also induce damage to the cell or organ and trigger ROS production (Valko et al., 2016; Tsai et al., 2020). The burst of ROS induced by the damaged mitochondria can trigger a series of adverse events that lead to tissue damage (Wang et al., 2020). Mitochondrial damage is a critical mechanism for exacerbating myocardial I/R injury (Wang et al., 2021; Wang et al., 2022). The iron reserve in the mitochondria accounts for approximately one-third of the total iron reserve in cardiomyocytes. The mitochondrial iron reserve was thus considered a major factor in determining cardiomyocyte fate (Wofford et al., 2017). Therefore, the protection of mitochondria against damage plays an important role in alleviating myocardial I/R injury, which may be an attractive strategy to suppress I/R injury (Wu et al., 2019; Altshuler et al., 2021). In this research, HJ11 decoction treatment attenuated mitochondrial damage and increased ATP and mtDNA levels in the myocardial I/R injury rat model and cell model. Mitochondria account for approximately 30% of the cardiac muscle cell volume, which produces approximately 95% of the total ATP to maintain the normal function of the heart (Xu et al., 2021). mtDNA plays an important role in self-replication, transcription, and coding proteins, which is independent of nuclear chromosomes. However, mtDNA in cardiomyocytes is susceptible to damage by ROS during myocardial I/R (Xu et al., 2021). Thus, HJ11 decoction might ameliorate myocardial I/R injury by suppressing ferroptosis-mediated mitochondrial damage.

Multiple genes participate in the ferroptosis process. These ferroptosis-associated genes can affect ROS production and lipid peroxidation both directly and indirectly, which finally results in oxidative cell death (Yan et al., 2021). GPX4 is a lipid repair enzyme, and its inactivation can cause damage to the intracellular antioxidant system, trigger ROS accumulation in mitochondria, and drive ferroptosis to induce ROS-mediated cell death (Yuan et al., 2016; Zhang et al., 2021). FTH1 is a critical subunit of ferritin that maintains cellular iron balance to prevent cells from ferroptosis (Zhao et al., 2021a). COX2 is a well-known biomarker of ferroptosis onset, and ACSL4 is a sensitive regulator of ferroptosis and a contributor to ferroptosis execution (Zhao et al., 2021b; Zhou et al., 2022). Interestingly, HJ11 decoction suppresses the expression of COX2 and ACSL4 and enhances the expression of FTH1 and GPX4 in the myocardial tissues of rats with I/R. In this way, HJ11 decoction might alleviate myocardial I/R injury by regulating the expression of these ferroptosis-related genes. A previous study has shown that traditional Chinese medicine (such as baicalin) can ameliorate myocardial I/R injury by suppressing ferroptosis through the inhibition of ACSL4 expression (Huang et al., 2019). In this research, the *in vitro* experiment indicated that HJ11 decoction alleviated ferroptosis-induced injury in OGD/R-induced H9c2 cells. More importantly, ACSL4 silencing attenuated ferroptosis in OGD/R-induced H9c2 cells, whereas ACSL4 overexpression abrogated the protective effect of HJ11 decoction on OGD/R-triggered ferroptosis in H9c2 cells. These findings imply that HJ11 decoction alleviated myocardial I/R injury by suppressing ACSL4-mediated ferroptosis. It is well known that apoptosis and ferroptosis are two different forms of cell death (Zhou et al., 2021). This study revealed that HJ11 decoction attenuated apoptosis and ferroptosis in the myocardial tissues of myocardial I/R injury rats. Interestingly, HJ11 decoction treatment induced a remarkable change in the regulation of lipid peroxidation, mitochondrial damage, and protein expression associated with ferroptosis. Therefore, inhibiting ferroptosis might be the most predominant mechanism by which HJ11 decoction alleviated myocardial I/R injury in rats. The *in vitro* experiment revealed that HJ11 decoction and Fer-1 had similar effects on OGD/R-induced H9c2 cells. All these findings suggested that HJ11 decoction might alleviate myocardial I/R injury by suppressing ferroptosis.

As expected, this study has limitations. First, the detailed ingredients and the HJ11 decoction doses used in humans are still in the confidential stage. It is not convenient to present this information in this paper. We will publish this information after the patent is granted. Second, this research suggested that ACSL4 is the major target of HJ11 to attenuate ferroptosis in the OGD/R-triggered cell model. More direct evidence should be provided to support this issue, such as binding or enzymatic assays. Additionally, it would be better to present *in vivo* data to strengthen the conclusion of HJ11 inhibiting ACSL4 to reduce ferroptosis. However, because of limitations in the laboratory equipment, these studies cannot be currently performed, which will be the focus of our future research.

## Conclusion

In summary, the findings of the present research suggest that HJ11 decoction exerted a protective effect against myocardial I/R injury and possibly alleviated myocardial I/R injury by inhibiting ACSL4-mediated ferroptosis. This is the first study wherein HJ11 decoction was proposed as a useful agent for the treatment of myocardial I/R injury. Further exploration of the underlying mechanisms, particularly ferroptosis, inflammation, and oxidative stress, needs to be carried out to provide a more solid basis for the clinical application of HJ11 decoction in the treatment of myocardial I/R injury.

## Data Availability

The original contributions presented in the study are included in the article/Supplementary Material, further inquiries can be directed to the corresponding authors.

## Supplementary material

SUPPLEMENTARY FIGURE S1 Baseline toxicity testing of high-dose HJ11 decoction administered to normal rats. Echocardiographic analysis of normal rats indicating that HJ11 decoction at a dose of 18 g/kg/d exerted no damage to the left ventricular anterior wall motion of rats. Hematoxylin-eosin staining showing that HJ11 decoction at a dose of 18 g/kg/d was nontoxic to the vital organs of normal rats, including heart, liver, spleen, lung, and kidney.

SUPPLEMENTARY FIGURE S2 Effects of HJ11 decoction and ACSL4 on oxidative stress and metabolic indicators in OGD/R-induced H9c2 cells **(A‐C)** ACSL4 overexpression abrogated the promoting effect of HJ11 decoction on the glutathione (rather than superoxide dismutase) level and the inhibitory effect of HJ11 decoction on the malondialdehyde level in oxygen glucose deprivation-reperfusion (OGD/R)–induced H9c2 cells. **(D,E)** ACSL4 overexpression reversed the promoting effect of HJ11 decoction on adenosine triphosphate (ATP) and mitochondrial DNA (mtDNA) levels in the OGD/R-induced H9c2 cells. **p* < 0.05. ***p* < 0.01. Data are shown as mean ± standard deviation. N = 3 for each condition.

## Abbreviations

I/R, Ischemia/reperfusion; AMI, Acute myocardial infarction; ACSL4, Acyl-CoA synthetase long-chain family member 4; COX2, Cyclooxygenase-2; FTH1, Ferritin heavy chain 1; GPX4, Glutathione-dependent lipid hydroperoxidase glutathione peroxidase 4; GSH, Glutathione; ROS, Reactive oxygen species; SOD, Superoxide Dismutase; MDA, Malonic dialdehyde; OGD/R:, Oxygen glucose deprivation/reperfusion; CCK-8, Cell Counting Kit-8 assay.
